# Robust sparse canonical correlation analysis

**DOI:** 10.1186/s12918-016-0317-9

**Published:** 2016-08-11

**Authors:** Ines Wilms, Christophe Croux

**Affiliations:** Leuven Statistics Research Centre (LStat), KU Leuven, Naamsestraat 69, Leuven, 3000 Belgium

**Keywords:** Canonical correlation analysis, Penalized estimation, Robust estimation

## Abstract

**Background:**

Canonical correlation analysis (CCA) is a multivariate statistical method which describes the associations between two sets of variables. The objective is to find linear combinations of the variables in each data set having maximal correlation. In genomics, CCA has become increasingly important to estimate the associations between gene expression data and DNA copy number change data. The identification of such associations might help to increase our understanding of the development of diseases such as cancer. However, these data sets are typically high-dimensional, containing a lot of variables relative to the number of objects. Moreover, the data sets might contain atypical observations since it is likely that objects react differently to treatments. We discuss a method for Robust Sparse CCA, thereby providing a solution to both issues. Sparse estimation produces canonical vectors with some of their elements estimated as exactly zero. As such, their interpretability is improved. Robust methods can cope with atypical observations in the data.

**Results:**

We illustrate the good performance of the Robust Sparse CCA method by several simulation studies and three biometric examples. Robust Sparse CCA considerably outperforms its main alternatives in (1) correctly detecting the main associations between the data sets, in (2) accurately estimating these associations, and in (3) detecting outliers.

**Conclusions:**

Robust Sparse CCA delivers interpretable canonical vectors, while at the same time coping with outlying observations. The proposed method is able to describe the associations between high-dimensional data sets, which are nowadays commonplace in genomics.

Furthermore, the Robust Sparse CCA method allows to characterize outliers.

**Electronic supplementary material:**

The online version of this article (doi:10.1186/s12918-016-0317-9) contains supplementary material, which is available to authorized users.

## Background

Canonical correlation analysis (CCA), introduced by [1], identifies and quantifies the associations between two sets of variables. CCA searches for linear combinations, called *canonical variates*, of each of the two sets of variables having maximal correlation. The coefficients of these linear combinations are called the *canonical vectors*. The correlations between the canonical variates are called the *canonical correlations*. CCA is used to study associations in, for instance, genomic data [2], environmental data [3], or biomedical data [4]. For more information on canonical correlations analysis, see e.g. [5], Chapter 10.

*Sparse* canonical vectors are canonical vectors with some of their elements estimated as exactly zero. The canonical variates then only depend on a subset of the variables, those corresponding to the non-zero elements of the estimated canonical vectors. Hence, the canonical variates are easier to interpret, in particular for high-dimensional data sets. Sparse estimation shows good performance in analyzing, for instance, genomic data (e.g. [6–8]), or biological data (e.g. [9, 10]). Examples of CCA for high-dimensional data sets can be found in, for example, genetics [11–13] and machine learning [14].

Different approaches for sparse CCA have been proposed in the literature. Parkhomenko et al. [15] use a sparse singular value decomposition to derive sparse singular vectors. Witten et al. [16] develop a penalized matrix decomposition, and show how to apply it for sparse CCA. Waaijenborg et al. [17], Lykou and Whittaker [18], An et al. [19] and Wilms and Croux [20] convert the CCA problem into a penalized regression framework to produce sparse canonical vectors. Chen et al. [21] and Gao and Zhou [22] discuss theoretical properties for sparse CCA. All these methods are not robust to outliers. A common problem in multivariate data sets, however, is the frequent occurrence of outliers. In genomics, for instance, some patients can react very differently to treatments because of their individual-specific genetic structure. Therefore, the possible presence of outlying observations should be taken into account.

Several *robust CCA* methods have been introduced in the literature. Dehon and Croux [23] considers robust CCA using the Minimum Covariance Determinant estimator [24]. Asymptotic properties for CCA based on robust estimators of the covariance matrix are discussed in [25]. Branco et al. [26] use a robust alternating regression approach to obtain the canonical variates. CCA can also be considered as a prediction problem, where the canonical variates obtained from the first data set serve as optimal predictors for the canonical variates of the second data set, and vice versa. As such, [27] use a robust M-scale to evaluate the prediction quality, whereas [28] use a robust estimator of the multivariate linear model. None of these methods, however, are sparse.

This paper proposes a CCA method that is sparse and robust at the same time. As such, we deal with two important topics in applied statistics: sparse model estimation and the presence of outliers in the data. We use an alternating robust, sparse regression framework to sequentially obtain the canonical variates. Robust Sparse CCA has clear advantages: (i) it provides well interpretable canonical vectors since some of the elements of the canonical vectors are estimated as exactly zero, (ii) it is still computable for high-dimensional data sets, where the sample size exceeds the number of variables in each data set, (iii) it can cope with outliers in the data, which are even more likely to occur in high dimensions, and (iv) it provides an interesting way to characterize these outliers.

Simulation studies were performed to investigate the performance of Robust Sparse CCA. These simulations show that Robust Sparse CCA achieves a substantially better performance compared to its leading alternatives CCA, Robust CCA and Sparse CCA. We illustrate the application of the Robust Sparse CCA method to an environmental data set and two genomic data sets. Robust Sparse CCA provides easy interpretable results. Moreover, we use Robust Sparse CCA to detect outlying observations in such high-dimensional data sets.

## Methods

First, we consider the robust and sparse estimator for the CCA problem. Next, we discuss the algorithm. Finally, we discuss the simulation designs and performance measures used to compare the performance of Robust Sparse CCA to standard CCA, Robust CCA and Sparse CCA.

### The estimator

We consider the CCA problem in a regression framework ([29, 30]). Given a sample of *n* observations $\mathbf {x}_{i} \in \mathbb {R}^{p}$ and $\mathbf {y}_{i} \in \mathbb {R}^{q}$ (*i*=1,…,*n*). The two data matrices are denoted as **X**=[**x**_1_,…,**x**_*n*_]^*T*^ and **Y**=[**y**_1_,…,**y**_*n*_]^*T*^. We assume the data matrices are robustly centered using the median. The estimated canonical vectors are collected in the columns of the matrices $\widehat {\mathbf {A}} \in \mathbb {R}^{p \times r}$ and $ \widehat {\mathbf {B}} \in \mathbb {R}^{q \times r}$. Here *r* is the number of canonical vectors. The columns of the matrices $\mathbf {X}\widehat {\mathbf {A}}$ and $\mathbf {Y}\widehat {\mathbf {B}}$ contain the estimates of the realizations of the canonical variates, and we denote their *j*^*th*^ column by $\hat {\mathbf {u}}_{j}$ and $\hat {\mathbf {v}}_{j}$, for 1≤*j*≤*r*. The objective function defining the canonical vector estimates is

1 $$ (\widehat{\mathbf{A}}, \widehat{\mathbf{B}}) = \underset{(\mathbf{A}, \mathbf{B}) }{\operatorname{argmin}} \ \sum_{i=1}^{n} || \mathbf{A}^{T}x_{i} - \mathbf{B}^{T} \mathbf{y}_{i} ||^{2}.  $$

The objective function in (1) is minimized under the restriction that each canonical variate $\hat {\mathbf {u}}_{j}$ is uncorrelated with the lower order canonical variates $\hat {\mathbf {u}}_{k}$, with 1≤*k*<*j*≤*r*. Similarly for the canonical vectors within the second set of variables. For identification purpose, a normalization condition requiring the canonical vectors to have unit norm is added. Typically, the canonical vectors are obtained by an eigenvalue analysis of a certain matrix involving the inverses of sample covariance matrices. But if *n*<max(*p,q*), these inverses do not exist.

We estimate the canonical vectors with an alternating regression procedure. If the matrix **A** in (1) is kept fixed, the matrix **B** can be obtained from a Least Squares regression of the canonical variates on **y** (and vice versa for estimating **A** keeping **B** fixed). The standard Least Squares estimator, however, is not sparse, nor robust to outliers. Therefore, we replace it by the sparse Least Trimmed Squares (sparse LTS) estimator [31]. The sparse LTS estimator can be applied to high-dimensional data and is robust to outliers.

### The algorithm

We use a sequential algorithm to derive the canonical vectors.

*First canonical vector pair.* Denote the first canonical vector pair by (**A**_1_,**B**_1_). Assume that the value of **A**_1_ is known. Denote the vector of squared residuals by $\mathbf {r}^{2}({{\mathbf {B}}_{1}})= \left ({r_{1}^{2}},\ldots,{r_{n}^{2}}\right)^{T}$, with ${r_{i}^{2}} = \left (\mathbf {A}_{1}^{T}\mathbf {x}_{i} - \mathbf {B}_{1}^{T} \mathbf {y}_{i}\right)^{2}, i=1,\ldots,n$. The estimate of **B**_1_ is obtained as

2 $$ \widehat{\mathbf{B}}_{1}|{\mathbf{A}}_{1} = \underset{\mathbf{B}_{1}}{\operatorname{argmin}} \sum_{i=1}^{h} \left(\mathbf{r}^{2}({{\mathbf{B}}_{1}}) \right)_{i:n} + h\lambda_{B_{1}} \sum_{j=1}^{q} |{b_{1}}_{j}|,   $$

where $\lambda _{B_{1}}>0$ is a sparsity parameter, *b*_1__*j*_ is the *j*^*th*^ element, *j*=1,…,*q*, of the first canonical vector **B**_1_, and (**r**^2^(**B**_1_))_1:*n*_≤…≤(**r**^2^(**B**_1_))_*n*:*n*_ are the order statistics of the squared residuals. The canonical vector $\widehat {\mathbf {B}}_{1}$ is normed to length 1. The solution to (2) equals the sparse LTS estimator with **XA**_1_ as response and **Y** as predictor. Regularization by adding a penalty term to the objective function is necessary since the design matrix **Y** can be high-dimensional. Sparse model estimates are obtained by adding an *L*_1_ penalty to the LTS objective function, similar as for the lasso regression estimator [32]. The sparse LTS estimator is computed with trimming proportion 25 %, so size of the subsample *h*=⌊0.75*n*⌋. To increase efficiency, we use a reweighting step. Further discussion and more detail on the sparse LTS estimator is provided in Additional file 1. As such, we get a robust sparse estimate $\widehat {\mathbf {B}}_{1}$.

Analogously, for a fixed value **B**_1_, denote the vector of squared residuals by $\mathbf {r}^{2}({{\mathbf {A}}_{1}})= ({r_{1}^{2}},\ldots,{r_{n}^{2}})^{T}$, with ${r_{i}^{2}} = \left (\mathbf {B}_{1}^{T}\mathbf {y}_{i} - \mathbf {A}_{1}^{T} \mathbf {x}_{i}\right)^{2}, i=1,\ldots,n$. The sparse LTS regression estimate of **A**_1_ with **YB**_1_ as response and **X** as predictor is given by

3 $$ \widehat{\mathbf{A}}_{1}|{\mathbf{B}}_{1} = \underset{\mathbf{A}_{1}}{\operatorname{argmin}} \sum_{i=1}^{h} \left(\mathbf{r}^{2}({{\mathbf{A}}_{1}}) \right)_{i:n} + h\lambda_{A_{1}} \sum_{j=1}^{p} |{a_{1}}_{j}|,   $$

where $\lambda _{A_{1}}>0$ is a sparsity parameter, *a*_1__*j*_ is the *j*^*th*^ element, *j*=1,…,*p* of the first canonical vector **A**_1_, and (**r**^2^(**A**_1_))_1:*n*_≤…≤(**r**^2^(**A**_1_))_*n*:*n*_ are the order statistics of the squared residuals. The canonical vector $\widehat {\mathbf {A}}_{1}$ is normed to length 1.

This leads to an alternating regression scheme, updating in each step the estimates of the canonical vectors until convergence.

After convergence of the algorithm, the values of **A**_1_ and **B**_1_ in subsequent iterations remain stable, and the same observations will be detected as outliers in regressions (2) and (3).

*Higher order canonical vector pairs.* We use deflated data matrices to estimate the higher order canonical vector pairs (see e.g. [26]). For the second canonical vector pair, the deflated matrices are $\mathbf {X}^{*}_{2}$, the residuals of a column-by-column LTS regression of **X** on all lower order canonical variates, $\hat {\mathbf {u}}_{1}$ in this case; and $\mathbf {Y}^{*}_{2}$, the residuals of a column-by-column LTS regression of **Y** on $\hat {\mathbf {v}}_{1}$. Since these regressions only involve a small number of regressors, the standard LTS estimator with *λ*=0 can be used.

The second canonical variate pair is then obtained by alternating between the following regressions until convergence:

4 $$ \widehat{\mathbf{B}}_{2}^{*}|{\mathbf{A}}^{*}_{2} = \underset{\mathbf{B}^{*}_{2}}{\operatorname{argmin}} \sum_{i=1}^{h} \left(\mathbf{r}^{2}({{\mathbf{B}}_{2}^{*}}) \right)_{i:n} + h\lambda_{B_{2}^{*}} \sum_{j=1}^{q} |{b_{2}^{*}}_{j}|,   $$

where $\mathbf {r}^{2}({{\mathbf {B}}_{2}^{\star }})= \left ({r_{1}^{2}},\ldots,{r_{n}^{2}}\right)^{T}$, with ${r_{i}^{2}} = \left (\mathbf {A}_{2}^{*T}\mathbf {x}_{2,i}^{\star } - \mathbf {B}_{2}^{\star T}\mathbf {y}_{2,i}^{\star }\right)^{2}, i=1,\ldots,n$.

5 $$ \widehat{\mathbf{A}}_{2}^{*}|{\mathbf{B}}^{*}_{2} = \underset{\mathbf{A}_{2}^{*}}{\operatorname{argmin}} \sum_{i=1}^{h} \left(\mathbf{r}^{2}({{\mathbf{A}}_{2}^{*}}) \right)_{i:n} + h\lambda_{A_{2}^{*}} \sum_{j=1}^{p} |{a_{2}^{*}}_{j}|,   $$

where $\mathbf {r}^{2}({{\mathbf {A}}_{2}^{\star }})= ({r_{1}^{2}},\ldots,{r_{n}^{2}})^{T}$, with ${r_{i}^{2}} = (\mathbf {B}_{2}^{*T}\mathbf {y}_{2,i}^{\star } - \mathbf {A}_{2}^{\star T} \mathbf {x}_{2,i}^{\star })^{2}, i=1,\ldots,n$. The canonical vectors $\widehat {\mathbf {B}}_{2}^{*}$ and $\widehat {\mathbf {A}}_{2}^{*}$ are both normed to length 1. We obtain $\hat {\mathbf {u}}^{*}_{2}=\mathbf {X}^{*}_{2}\widehat {\mathbf {A}}_{2}^{*}$ and $\hat {\mathbf {v}}^{*}_{2}=\mathbf {Y}^{*}_{2}\widehat {\mathbf {B}}_{2}^{*}$.

Finally, the second canonical vector needs to be expressed as linear combinations of the columns of the original data matrices, and not the deflated ones. Since we want to allow for zero coefficients in these linear combinations, a sparse approach is needed. To obtain a sparse $\widehat {\mathbf {A}}_{2}$, we regress $\hat {\mathbf {u}}^{*}_{2}$ on **X** using the sparse LTS estimator, yielding the fitted values $\hat {\mathbf {u}}_{2}=\mathbf {X}\widehat {\mathbf {A}}_{2}$. To obtain a sparse $\widehat {\mathbf {B}}_{2}$, we regress $\hat {\mathbf {v}}^{*}_{2}$ on **Y** using the sparse LTS estimator, yielding the fitted values $\hat {\mathbf {v}}_{2}=\mathbf {Y}\widehat {\mathbf {B}}_{2}$.

The higher order canonical variate pairs are obtained in a similar way. We perform alternating sparse LTS regressions as in (4) and (5), followed by a final sparse LTS step to retrieve the estimated canonical vectors $({\widehat {\mathbf {A}}}_{l},{\widehat {\mathbf {B}}}_{l})$. It is not really necessary to use a sparse approach in regressions (4) and (5), other penalty functions can be used.

A schematic representation of the complete algorithm is provided in Additional file 2.

Finally, note that as in other sparse CCA proposals (e.g. [15–17, 20]) the canonical variates are in general not uncorrelated. The robust sparse canonical vectors we obtain yield an interpretable basis of the space spanned by the canonical vectors. This basis can be made orthogonal (but not sparse) after suitable rotation if one desires so.

*Initial value.* A starting value for **A**_1_ is required to start up the algorithm. We compute the first robust principal component of **Y**, denoted **z**_1_. The first robust principal component is calculated from the first eigenvector of the robustly estimated covariance matrix. For this aim, we use the spatial sign covariance estimator [33]. We regress **z**_1_ on **X** using the sparse LTS. The estimated regression coefficient matrix of this regression is used as initial value for **A**_1_. To obtain an initial estimate for the higher order canonical vectors **A**_*l*_, for *l*=2,…,*r*, we use the first robust principal component of the deflated data matrix and proceed analogously.

We performed several numerical experiments to investigate the sensitivity of the outcome of the algorithm to the choice of initial value. In low-dimensional settings, the choice of initial value is not important. In high-dimensional settings, a good initial value is more important. Note that the initial value should exist and be easily computable in all settings, which holds for our proposal.

*Number of canonical variates to extract.* To decide on the number of canonical variates *r* to extract, we use the maximum eigenvalue ratio criterion of [19]. We apply the Robust Sparse CCA algorithm and calculate the robust correlations $\hat {\rho }_{1}, \ldots,\hat {\rho }_{\text {rmax}} $, with rmax=min(*p,q*,10). For high-dimensional data sets, we consider a maximum of 10 canonical correlations, since in practice, more than 10 canonical vector pairs are never used. Each $\hat \rho _{j}$ is obtained by computing the correlation between $\hat {\mathbf {v}}_{j}$ and $\hat {\mathbf {u}}_{j}$ from the bivariate Minimum Covariance Determinant (MCD) estimator with 25 % trimming. Let $\hat {k}_{j} = \hat {\rho }_{j}/\hat {\rho }_{j+1}$ for *j*=1,…,rmax−1. We extract *r* pairs of canonical variates, where $r=\text {argmax}_{j} \hat {k}_{j}$.

*Convergence criterion.* In each step of the alternating regression algorithm we update the estimates of the canonical vectors $\widehat {\mathbf {B}}_{l}^{*} $ and $\widehat {\mathbf {A}}_{l}^{*} $, for *l*=1,…,*r*. We iterate until the relative change in the value of the convergence criterion in two successive iterations^1^ is smaller than the convergence tolerance value *ε*=10^−2^. As convergence criterion, we consider

$$ \text{Convergence criterion} = \frac{1}{h} \sum_{i=1}^{h} \left(\mathbf{r}^{2}\left(\widehat{{\mathbf{A}}}_{l}^{*}, \widehat{{\mathbf{B}}}_{l}^{*} \right) \right)_{i:n},   $$

for *l*=1,…,*r*, where $\mathbf {r}^{2}\left (\widehat {{\mathbf {A}}}_{l}^{*}, \widehat {{\mathbf {B}}}_{l}^{*}\right)= \left ({r_{1}^{2}},\ldots,{r_{n}^{2}}\right)^{T}$, with ${r_{i}^{2}} = \left (\widehat {{\mathbf {A}}}_{l}^{*T}\mathbf {x}_{l,i}^{\star } - \widehat {{\mathbf {B}}}_{l}^{*T} \mathbf {y}_{l,i}^{\star }\right)^{2}, i=1,\ldots,n$. $\mathbf {X}^{*}_{l}$ and $\mathbf {Y}^{*}_{l}$ are the original data sets for *l*=1, and the deflated data matrices for *l*=2,…,*r*. In the simulations we conducted, convergence was almost always reached.^2^ For data sets with *n*=100,*p*=*q*=10, on average 6 iterations per canonical vector pair are needed to converge. For *n*=50,*p*=*q*=100, on average 10 iterations are needed to converge.

*Choice of the sparsity parameter.* The sparsity parameters controlling the penalization on the regression coefficient matrices are selected with the Bayesian Information Criterion (e.g. [34]). We use a range of values for the sparsity parameters and select the one with the lowest value of

$$ \text{BIC}_{\lambda_{\widehat{\mathbf{A}}^{*}_{l}}} = n \cdot\text{log}\left(\frac{1}{h} \sum_{i=1}^{h} \left(\mathbf{r}^{2}({{\widehat{\mathbf{A}}}_{l}^{*}}) \right)_{i:n} \right) + df_{\lambda_{\widehat{\mathbf{A}}^{*}_{l}}} \cdot \log(n),   $$

$$ \text{BIC}_{\lambda_{\widehat{\mathbf{B}}^{*}_{l}}} = n \cdot\text{log}\left(\frac{1}{h} \sum_{i=1}^{h} \left(\mathbf{r}^{2}({\widehat{\mathbf{B}}_{l}^{*}}) \right)_{i:n} \right) + df_{\lambda_{\widehat{\mathbf{B}}^{*}_{l}}} \cdot \log(n),   $$

for *l*=1,…,*r*, with $df_{\lambda _{\widehat {\mathbf {A}}^{*}_{l}}}$ and $df_{\lambda _{\widehat {\mathbf {B}}^{*}_{l}}}$ the respective number of non-zero estimated regression coefficients.

*Computation time.* All computations are carried out in **R** version 3.2.1. The code of the algorithm is made available on a webpage of the first author (http://feb.kuleuven.be/ines.wilms/software). For data sets with *n*=100,*p*=*q*=10, on average 10 seconds are needed to extract one canonical vector pair on an Intel Xeon E5-2699 v3 @ 2.30GHz machine. For *n*=50,*p*=*q*=100, we need 540 seconds on average, for *n*=100,*p*=*q*=10000, computation time increases to 11 hours on average. But even in high dimensions, the number of iterations remains lows (8 on average). The high computing time needed for *p*=*q*=10000 is mainly due to the sparse LTS estimator, taken from the **R**-package **robustHD** [35]. By including a variable screening step [36] preceding the computation of the sparse LTS estimator, one could reduce the total computation time considerably.

### Simulation designs

To investigate the performance of Robust Sparse CCA, we conduct a simulation study. We consider several simulation designs.

In the “Uncorrelated Sparse Low-dimensional” and “Correlated Sparse Low-dimensional” design, there is one canonical variate pair and the canonical vectors have a sparse structure. The variables within each data set are uncorrelated in the first design, and correlated in the second design. In the “NonSparse Low-dimensional” design, there are two canonical variate pairs and the canonical vectors are non-sparse. The remaining three designs are high-dimensional with a lot of variables compared to the sample size. Only Sparse CCA and Robust Sparse CCA can be computed. In the “Sparse High-dimensional 1” design with *n*=100,*p*=100,*q*=4, there is one canonical variate pair and the canonical vectors are sparse. In the “Sparse High-dimensional 2” design with *n*=100,*p*=*q*=100, there is one canonical variate pair and each canonical vector contains ten non-zero elements. In the “Sparse Ultra High-dimensional” design there are much more variables (*p*=*q*=10000) than observations (*n*=100). There is one canonical variate pair and each canonical vector contains ten non-zero elements. The number of simulations for each design except the last one is *M*=1000. For the “Sparse Ultra High-dimensional design” *M*=100 to reduce computational burden.

For each design, the following settings are considered

- *No contamination.* We generate data matrices **X** and **Y** according to a multivariate normal distribution *N*_*p*+*q*_(**0**,**Σ**), with covariance matrix
  $$ {\boldsymbol{\Sigma}} = \left[\begin{array}{ll} {\boldsymbol{\Sigma}}_{xx} & {\boldsymbol{\Sigma}}_{xy} \\{\boldsymbol{\Sigma}}_{xy}^{T} & {\boldsymbol{\Sigma}}_{yy} \end{array}\right]   $$
  described in Table 1.

**Table 1:** Simulation designs

**Design**	***Σ***_*xx*_	***Σ***_*yy*_	***Σ***_*xy*_
Uncorrelated Sparse Low-dimensional	10^−2^·**I**_*p*_	10^−2^·**I**_*q*_	$10^{-2}\cdot \left [\begin {array}{ll} 0.9 & {\mathbf {0}}_{1 \times 3} \\ {\mathbf {0}}_{5 \times 1} & {\mathbf {0}}_{5 \times 3} \end {array}\right ]$
*n*=100,*p*=6,*q*=4			
Correlated Sparse Low-dimensional	$10^{-2}\cdot \left [\begin {array}{ccc} 1 & 0.4 & \mathbf {0}\\ 0.4 & 1 & \mathbf {0}\\ 0 & 0 & \mathbf {I}_{4\times 4} \end {array}\right ]$	$10^{-2}\cdot \left [\begin {array}{ccc} 1 & 0.4 & \mathbf {0}\\ 0.4 & 1 & \mathbf {0}\\ 0 & 0 & \mathbf {I}_{2\times 2} \end {array}\right ]$	$ 10^{-2}\cdot \left [\begin {array}{cc} 0.8 & {\mathbf {0}}_{1 \times 3} \\ {\mathbf {0}}_{5 \times 1} & {\mathbf {0}}_{5 \times 3} \end {array}\right ]$
*n*=100,*p*=6,*q*=4			
NonSparse Low-dimensional	10^−2^·**I**_*p*_	10^−2^·**I**_*q*_	10^−2^·**0.1**_*p*×*q*_
*n*=100,*p*=12,*q*=8			
Sparse High-dimensional 1	10^−1^·**I**_*p*_	10^−1^·**I**_*q*_	$10^{-1} \cdot \left [\begin {array}{cc}{\mathbf {0.45}}_{2 \times 2} & {\mathbf {0}}_{2 \times 2} \\ {\mathbf {0}}_{98 \times 2} & {\mathbf {0}}_{98 \times 2} \end {array}\right ]$
*n*=100,*p*=100,*q*=4			
Sparse High-dimensional 2	$10^{-7} \cdot \left [\begin {array}{cc} \mathbf {S}_{10\times 10} & \mathbf {0} \\ \mathbf {0} & 10^{-3}\cdot \mathbf {I}_{90\times 90} \\ \end {array}\right ]$	***Σ***_*xx*_	$10^{-7}\cdot \left [\begin {array}{cc}\mathbf {0.8}_{10\times 10} & {\mathbf {0}}_{10 \times 90} \\ {\mathbf {0}}_{90 \times 10} & {\mathbf {0}}_{90 \times 90} \end {array}\right ]$
*n*=50,*p*=*q*=100			
	with $\mathbf {S}_{ij}= \left \{\begin {array}{ll} 1 & if \ i=j\\ 0.8 & if \ i\neq j, \end {array}\right.$		
Sparse Ultra High-dimensional	$10^{-7} \cdot \left [\begin {array}{cc} \mathbf {S}_{10\times 10} & \mathbf {0} \\ \mathbf {0} & 10^{-3}\cdot \mathbf {I}_{9990\times 9990} \\ \end {array}\right ]$	***Σ***_*xx*_	$10^{-7}\cdot \left [\begin {array}{cc}\mathbf {0.8}_{10\times 10} & {\mathbf {0}}_{10 \times 9990} \\ {\mathbf {0}}_{9990 \times 10} & {\mathbf {0}}_{9990 \times 9990} \end {array}\right ]$
*n*=100,*p*=*q*=10000			
	with $\mathbf {S}_{ij}= \left \{\begin {array}{ll} 1 & if \ i=j\\ 0.8 & if \ i\neq j, \end {array}\right.$
- *t-distribution.* We generate data matrices **X** and **Y** according to a multivariate *t*-distribution with three degrees of freedom *t*_3_(**0**,**Σ**).
- *Contamination.* 90 % of the data are generated from *N*_*p*+*q*_(**0**,**Σ**), and 10 % of the data are generated from *N*_*p*+*q*_(**2**,*Σ*_cont_), with
  $$ {\boldsymbol\Sigma_{\text{cont}}} = \left[\begin{array}{ccc} \boldsymbol \Sigma_{xx} && \mathbf{0} \\ \mathbf{0} && \boldsymbol \Sigma_{yy} \end{array}\right].   $$
  Similar conclusions can be drawn from other contamination settings (e.g. where only one of the two data sets is contaminated) and are available from the authors upon request.

### Performance measures

In our simulation study, the estimators are evaluated on their estimation accuracy and sparsity recognition performance.

For evaluating estimation accuracy, we compute for each simulation run *m*, with *m*=1,…,*M*, the angle $\theta ^{m}(\hat {\mathbf {A}}^{m},\mathbf {A})$ between the subspace spanned by the estimated canonical vectors (contained in the columns of $\hat {\mathbf {A}}^{m}$) and the subspace spanned by the true canonical vectors (contained in the columns of **A**). We proceed analogously for the matrix **B**. The average angles, measuring the estimation accuracy, are given by

$$\begin{aligned} \bar{\theta}(\hat{\mathbf{A}},\mathbf{A}) &= \frac{1}{M} \sum_{m=1}^{M} \theta^{m}(\hat{\mathbf{A}}^{m},\mathbf{A}) \text{\ \ \ and \ \ \ \ }\\ \bar{\theta}(\hat{\mathbf{B}},\mathbf{B}) &= \frac{1}{M} \sum_{m=1}^{M} \theta^{m}(\hat{\mathbf{B}}^{m},\mathbf{B}). \end{aligned} $$

For evaluating sparsity, we use the true positive rate and the true negative rate

$$ \begin{aligned} \text{TPR}(\hat{\mathbf{A}}^{m},\mathbf{A}) &= \frac{\# \{ (i,j):{{\widehat{\mathbf{A}}_{ij}}}^{m} \neq 0 \text{and} {\mathbf{A}}_{ij}\neq 0 \}}{ \# \{ (i,j): {\mathbf{A}}_{ij} \neq 0 \} }\\ \text{TNR}(\hat{\mathbf{A}}^{m},\mathbf{A}) &= \frac{\# \{ (i,j):{ {\widehat{\mathbf{A}}_{ij}}}^{m}= 0 \text{and}{\mathbf{A}}_{ij} = 0 \}} {\# \{ (i,j): {\mathbf{A}}_{ij} = 0\}}. \end{aligned} $$

We proceed analogously for the matrix **B**. A true positive is a coefficient that is non-zero in the true model, and is estimated as non-zero. A true negative is a coefficient that is zero in the true model, and is estimated as zero. Both should be as high as possible for a sparse estimator. Note that the false positive rate is the complement of the true negative rate (i.e. FPR=1-TNR). A sparse estimator should control the FPR, which can be seen as a false discovery rate, at a sufficiently low level.

In our empirical applications, to decide on the number of canonical variate pairs to extract, we use the maximum eigenvalue ratio criterion, as discussed in the “Methods” Section. To compare the performance of the CCA approaches, we perform a leave-one-out cross-validation exercise and compute the cross-validation score

6 $$ CV = \frac{1}{r} \frac{1}{h} \sum_{i=1}^{h} || \widehat{\mathbf{A}}^{T}_{-i}\mathbf{x}_{i} - \widehat{\mathbf{B}}^{T}_{-i}\mathbf{y}_{i} ||^{2},   $$

where $\widehat {\mathbf {A}}^{T}_{-i}$ and $\widehat {\mathbf {B}}^{T}_{-i}$ contain the estimated canonical vectors when the *i*^*th*^ observation is left out of the estimation sample and *h*=⌊*n*(1−*α*)⌋, with *α*=0 (0 % Trimming) or *α*=0.1 (10 % Trimming). We use trimming to eliminate the effect of outliers in the cross-validation score.

## Results

### Simulation study

We compare the performance of the Robust Sparse CCA method with (i) standard CCA, (ii) Robust CCA, and (iii) Sparse CCA. The alternating regression algorithm is used for all four estimators, for ease of comparability. Robust CCA uses LTS instead of sparse LTS, and corresponds to the alternating regression approach of [26]. Sparse CCA uses the lasso instead of sparse LTS, Pearson correlations for computing the canonical correlations, and ordinary PCA for getting the initial values. The sparsity parameters for sparse CCA are selected with BIC. Standard CCA is like sparse CCA, but using the LS instead of the lasso.

Summary results for the estimator $\widehat {\mathbf {A}}$ are in Table 2. The results for $\widehat {\mathbf {B}}$ are similar and, therefore, omitted. Standard errors around the average angles, TPRs and TNRs are in almost all cases smaller than 6 % of the reported numbers in Table 2.

**Table 2:** Simulation results. Average of the angles between the space spanned by the true and estimated canonical vectors; average true positive rate and true negative rate are reported for each method

Design	Method	No contamination			*t*-distribution			Contamination		
		$\bar {\theta }(\hat {\mathbf {A}},\mathbf {A})$	TPR	TNR	$\bar {\theta }(\hat {\mathbf {A}},\mathbf {A})$	TPR	TNR	$\bar {\theta }(\hat {\mathbf {A}},\mathbf {A})$	TPR	TNR
Uncorrelated	CCA	0.11	1.00	0.00	0.22	1.00	0.00	0.38	1.00	0.00
Sparse	Robust CCA	0.14	1.00	0.00	0.15	1.00	0.00	0.15	1.00	0.00
Low-dimensional	Sparse CCA	0.04	0.98	0.97	0.19	0.94	0.63	0.34	1.00	0.04
	Robust Sparse CCA	0.04	1.00	0.82	0.11	1.00	0.52	0.05	1.00	0.76
Correlated	CCA	0.06	1.00	0.00	0.13	1.00	0.00	0.43	1.00	0.00
Sparse	Robust CCA	0.08	1.00	0.00	0.09	1.00	0.00	0.09	1.00	0.00
Low-dimensional	Sparse CCA	0.13	1.00	1.00	0.19	0.96	0.76	0.57	0.52	0.02
	Robust Sparse CCA	0.07	1.00	0.57	0.09	1.00	0.34	0.07	1.00	0.53
NonSparse	CCA	0.08	1.00	NA	0.32	1.00	NA	0.20	1.00	NA
Low-dimensional	Robust CCA	0.11	1.00	NA	0.12	1.00	NA	0.12	1.00	NA
	Sparse CCA	0.41	0.93	NA	0.67	0.82	NA	0.23	1.00	NA
	Robust Sparse CCA	0.16	0.99	NA	0.22	0.99	NA	0.13	1.00	NA
Sparse	Sparse CCA	0.65	0.62	0.99	0.70	0.71	0.87	0.36	1.00	0.80
High-Dimensional 1	Robust Sparse CCA	0.66	0.84	0.86	0.56	0.82	0.86	0.16	0.96	0.97
Sparse	Sparse CCA	1.08	0.31	1.00	1.14	0.23	1.00	1.25	0.38	0.97
High-Dimensional 2	Robust Sparse CCA	0.59	0.87	0.87	0.60	0.94	0.89	0.84	0.97	0.82
Sparse Ultra	Sparse CCA	1.18	0.17	1.00	1.22	0.15	1.00	1.25	0.40	1.00
High-dimensional	Robust Sparse CCA	1.42	0.93	1.00	1.24	0.98	1.00	0.98	1.00	1.00

First we discuss the results from the “Uncorrelated Sparse Low-dimensional” design. In the scenario without contamination, the sparse estimators Sparse CCA and Robust Sparse CCA achieve a much better average estimation accuracy than the non-sparse estimators CCA and Robust CCA. As expected, a sparse method results in increased estimation accuracy when the true canonical vectors have a sparse structure. Looking at sparsity recognition performance, Sparse CCA and Robust Sparse CCA perform equally good in retrieving the sparsity in the data generating process. In the contaminated simulation setting, the robust estimators maintain their accuracy. Robust Sparse CCA performs best and clearly outperforms Robust CCA: for instance, Robust Sparse CCA achieves an average estimation accuracy of 0.05 against 0.15 for the contamination setting, see Table 2. The non-robust estimators CCA and Sparse CCA are clearly influenced by the outliers, as reflected by the much higher values of the average angle $\bar {\theta }(\hat {\mathbf {A}},\mathbf {A})$ in Table 2. Sparse CCA now performs even worse than Robust CCA. The considered contamination induces overfitting in Sparse CCA, reflected in the low values of the true negative rate, or alternatively, the high values of the false positive rate.

In an unreported simulation study, we investigated the effect of the signal strength on the results. We vary the value of the true canonical correlation in the first design from 0.1 to 0.9, thereby increasing the signal strength. If outliers are present, Robust Sparse CCA always performs best. The margin by which it outperforms Sparse CCA is larger if the signal is stronger. If no outliers are present, Sparse CCA performs best for weak signal levels below 0.6.

Similar conclusions can be drawn from the “Correlated Sparse Low-dimensional” and “NonSparse Low-dimensional” design. Note that the true negative rate in Table 2 is omitted for the “NonSparse Low-dimensional” design since the true canonical vectors are non-sparse. In the situation without contamination, the price the sparse methods pay in the “NonSparse Low-dimensional” design is a decreased estimation accuracy, as measured by the average angle. For Robust Sparse CCA compared to Robust CCA this decrease is marginal. In the contaminated settings, the robust methods perform best and show similar performance.

For the high-dimensional designs, only Sparse CCA and Robust Sparse CCA are computable. For the “Sparse High-dimensional 1” design, Robust Sparse CCA is competitive to Sparse CCA if no outliers are present. When adding outliers, the performance of Sparse CCA gets distorted. For the heavier tailed *t*-distribution, the average estimation accuracy of Robust Sparse CCA compared to Sparse CCA is much better: 0.56 against 0.70. For the contamination setting, the average estimation accuracy of Robust Sparse CCA is even more than twice as good as the average estimation accuracy of Sparse CCA. Similar conclusions hold for the second high-dimensional design.

In the “Sparse Ultra High-dimensional” design, Sparse CCA performs best if no outliers are present. For the heavier tailed *t*-distribution, Robust Sparse CCA and Sparse CCA perform comparable in terms of estimation accuracy. But in the presence of outliers, Robust Sparse CCA improves estimation accuracy of Sparse CCA by about 22 %. Moreover, Robust Sparse CCA achieves a good balance between the TPR and the TNR, while Sparse CCA suffers from a low TPR if outliers are present.

In sum, Robust Sparse CCA shows the best overall performance in this simulation study. It performs best in sparse contaminated settings. In sparse non-contaminated settings, Robust Sparse CCA is competitive to Sparse CCA. In contaminated non-sparse settings, Robust Sparse CCA is competitive to Robust CCA.

### Comparison of Robust Sparse CCA to other CCA alternatives

We compare the performance of Robust Sparse CCA to

- the sparse CCA methods of [15, 16] and [17]. The sparsity parameters of all methods are selected as proposed by the respective authors. Note that these methods are not robust.
- sparse CCA applied on pre-processed data. As a pre-processing step to remove outliers, we transformed the data towards normality by replacing them by their normal scores (see e.g. [37], page 150).
- sparse CCA using the robust initial value for the algorithm as Robust Sparse CCA.

Summary results for the estimator $\widehat {\mathbf {A}}$ are in Table 3. For reasons of brevity, we only report the results from the “Sparse High-dimensional 2” design. Similar conclusions are obtained from the other designs and are available from the authors upon request.

**Table 3:** As in Table 3, comparing Robust Sparse CCA to other alternatives in the “Sparse High-dimensional 2 design”

Method	No contamination			*t*-distribution			Contamination		
	$\bar {\theta }(\hat {\mathbf {A}},\mathbf {A})$	TPR	TNR	$\bar {\theta }(\hat {\mathbf {A}},\mathbf {A})$	TPR	TNR	$\bar {\theta }(\hat {\mathbf {A}},\mathbf {A})$	TPR	TNR
Sparse CCA of [15]	0.93	1.00	0.93	1.41	0.94	0.72	1.28	0.89	0.00
Sparse CCA of [16]	0.79	0.65	1.00	1.16	0.30	0.92	1.57	0.00	0.00
Sparse CCA of [17]	0.44	1.00	0.08	1.01	1.00	0.02	1.25	1.00	0.00
Sparse CCA on pre-processed data	0.58	0.92	0.79	0.72	0.88	0.74	1.36	0.74	0.25
Sparse CCA with robust initialization	1.07	0.32	1.00	1.13	0.24	1.00	1.25	0.38	0.97
Robust Sparse CCA	0.59	0.87	0.87	0.60	0.94	0.89	0.84	0.97	0.82

If no outliers are present, (i) Robust Sparse CCA is competitive to the sparse CCA methods of [15, 16] and [17]. (ii) Robust Sparse CCA performs comparable to Sparse CCA on pre-processed data. (iii) Sparse CCA with the same initial value as Robust Sparse CCA performs comparable to Sparse CCA.

If outliers are present, (i) Robust Sparse CCA outperforms the sparse CCA methods of [15, 16] and [17]. (ii) Robust Sparse CCA outperforms Sparse CCA on pre-processed data. Sparse CCA on pre-processed data performs better than Sparse CCA. (iii) Robust Sparse CCA outperforms Sparse CCA with the same initial value. Here, differences in performance between Robust Sparse CCA and Sparse CCA stem from the use of the sparse LTS instead of the lasso regressions. Hence, the use of the sparse LTS estimator in the alternating regression scheme is essential.

### Applications

We consider three biometric applications. The first data set is low-dimensional and often used in Robust Statistics. The other two data sets are high-dimensional and have been used before in papers on sparse CCA. We show that the performance of Robust Sparse CCA on these data sets is much better than the performance of Sparse CCA.

#### Evaporation data set

We analyze an environmental data set from [38]. Two sets of environmental variables have been measured on *n*=46 consecutive days from June 6 until July 21.^3^ The first set contains *p*=3 soil temperature variables (maximum, minimum and average soil temperature). The second set contains *q*=7 environmental variables (maximum, minimum and average air temperature; maximum, minimum and average daily relative humidity; and total wind). The aim is to find and quantify the relations between the soil temperature variables and the remaining variables.

**Fig. 1:**
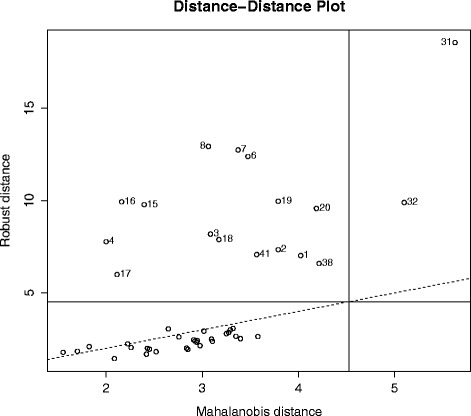
Evaporation data set: Distance-Distance plot

As a first inspection of the data, we use the Distance-Distance plot [39] in Fig. 1. The Distance-Distance plot displays the robust distances versus the Mahalanobis distances. The vertical and horizontal lines are drawn at values equal to the square root of the 97.5 % quantile of a chi-squared distribution with 10 degrees of freedom. Points beyond those lines would be considered as outliers. The Distance-Distance plot reveals some outliers: objects 31 and 32, for example, are extreme outliers. This suggests the need for a robust CCA method. Table 4 reports the cross-validation scores from Eq. (6) for the four CCA methods. For all methods two canonical variate pairs are extracted. The method that achieves the lowest cross-validation score has the best out-of-sample performance. Robust Sparse CCA achieves the best cross-validation score.

**Table 4:** Evaporation data set: Cross-validation score for standard CCA, Robust CCA, Sparse CCA and Robust Sparse CCA

Method			CV-score		CV-score
			0 % Trimming		10 % Trimming
CCA			0.74		0.49
Robust CCA			0.57		0.39
Sparse CCA			0.57		0.41
Robust Sparse CCA			0.48		0.31

Table 5 shows the estimated canonical vectors for the Robust CCA and Robust Sparse CCA method. By adding the penalty term, the number of non-zero coefficients in the two canonical vectors is reduced from a total of 20 for Robust CCA to 10 for Robust Sparse CCA. The price to pay for the sparseness is a slight decrease in the estimated canonical correlations (computed using the bivariate MCD estimator, see “Methods” Section): they drop from 0.93 to 0.87 for the first one, and from 0.56 to 0.48 for the second canonical correlation. We find this decrease acceptable, given the gained sparsity in the canonical vectors. The sparse structure of the canonical vectors facilitates interpretation. The first canonical variate in the soil temperature data set, for instance, is uniquely determined by the variable AVST.

**Table 5:** Evaporation data set: Estimated canonical vectors using Robust CCA and Robust Sparse CCA

		Robust CCA		Robust Sparse CCA	
	Variables ∖Canonical Vectors	1	2	1	2
First	MAXST: Max. daily soil temperature	–0.35	–0.76	0	–0.70
data	MINST: Min. daily soil temperature	0.03	0.63	0	0.71
set	AVST: Avg. daily soil temperature	0.93	0.18	1	0
Second	MAXAT: Max. daily air temperature	0.54	–0.11	0.94	0
data	MINAT: Min. daily air temperature	0.67	0.84	0.14	0.38
set	AVAT: Avg. daily air temperature	0.14	–0.03	0.17	0.36
	MAXH: Max. daily relative humidity	–0.13	0.09	0	0
	MINH: Min. daily relative humidity	–0.03	0.36	0	0.85
	AVH: Avg. daily relative humidity	–0.28	0.32	–0.24	0
	WIND: Total wind, measured in miles per day	–0.37	–0.19	0	0
	*Canonical correlations*	0.93	0.56	0.87	0.48

#### Nutrimouse data set

This genetic data set is publicly available in the R package **CCA** [11]. Two sets of variables, i.e. gene expressions and fatty acids, are available for *n*=40 mice. The first set contains expressions of *p*=120 genes measured in liver cells. The second set of variables contains concentrations of *q*=21 hepatic fatty acids (FA). In this experiment, there are two groups of mice (wild-type and PPAR *α* deficient mice) that receive a specific diet (five possible diets). More details on how the data were obtained can be found in [40]. The aim is to identify a small set of genes that are correlated with the fatty acids.

In this data set, the number of experimental units is smaller than the number of variables. Therefore, standard CCA nor Robust CCA can be performed. Robust Sparse CCA and Sparse CCA can be applied in this high-dimensional setting and produce interpretable, sparse canonical vectors. For both methods, one canonical variate pair is extracted. The cross-validation scores from Eq. (6) are reported in Table 6. Robust Sparse CCA outperforms Sparse CCA. The cross-validation scores are reduced by about 90 % when using the robust method.

**Table 6:** Nutrimouse data set: Cross-validation score for Sparse CCA and Robust Sparse CCA

Method	CV-score	CV-score
	0 % Trimming	10 % Trimming
Sparse CCA	98.78	92.53
Robust Sparse CCA	6.30	4.31

Given its better out-of-sample performance, we discuss the estimated canonical vectors obtained using Robust Sparse CCA.

**Fig. 2:**
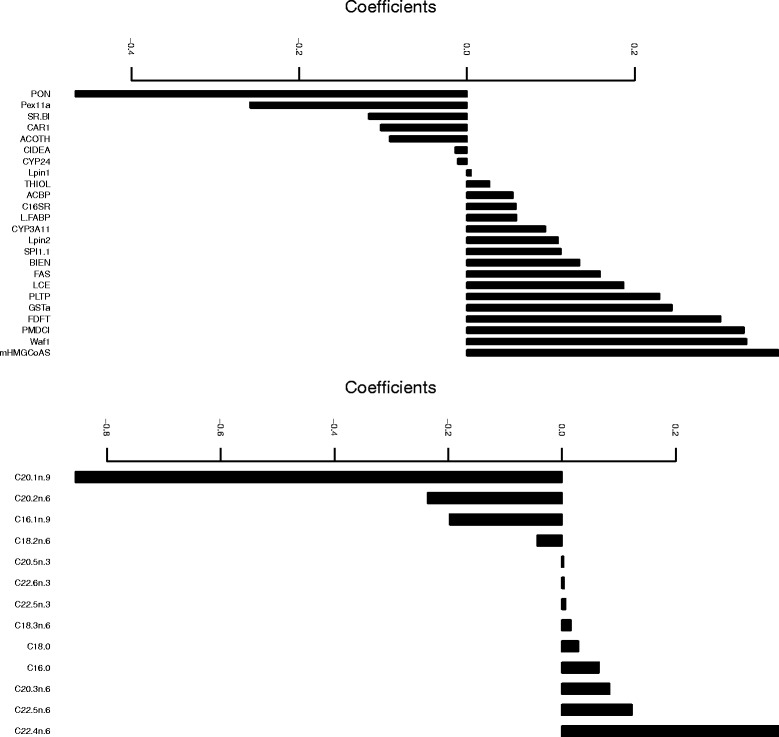
Nutrimouse data set: Coefficients of selected genes (*top*) and coefficients of selected fatty acids (*bottom*) in the first canonical vector pair

The top panel of Fig. 2 displays the coefficients of the selected genes, i.e. those genes with non-zero estimated coefficients, in the first canonical vector: 24 out of 120 variables are selected. The solution is very sparse, facilitating interpretation. Martin et al. [40] find a consistent reduction of **Cyp3a11** in PPAR *α* livers on the one hand, and an overexpression of **CAR1** on the other hand. Both genes are selected and have among the highest (absolute) coefficients. The coefficients of the selected fatty acids are displayed in the bottom panel of Fig. 2: 13 out of 21 fatty acid variables are selected. The fatty acids **C22:6n-3**, **C22:5n-3**, **C22:5n-6**, **C22:4n-3** and **C20:5n-3** are related to the effect of the five diets used in this experiment. From Fig. 2, we see that four out of these five fatty acids are selected.

#### Breast cancer data set

The genetic data set is described in [41] and available in the R package PMA [42]. Two sets of data, i.e. gene expression data (19 672 variables) and comparative genomic hybridization (CGH) data (2149 variables) are available for *n*=89 patients, and this for 23 chromosomes. We analyze the data for each of the chromosomes separately, each time using the CGH and gene expression variables for that particular chromosome. Depending on the chromosome, either 1, 2, 3, or 4 canonical vector pairs are extracted. The aim is to identify a subset of CGH variables that are correlated with a subset of gene expression variables.

**Fig. 3:**
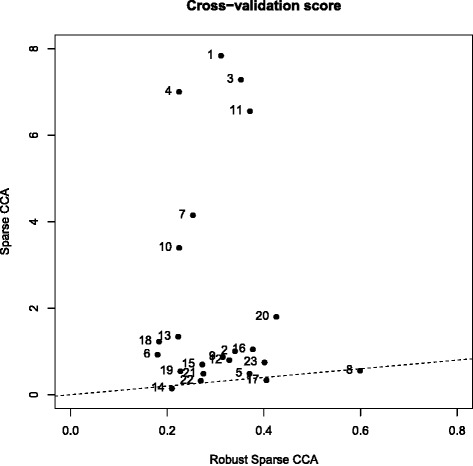
Breast cancer data set: 23 cross-validation scores (one for each chromosome) for Robust Sparse CCA (*horizontal axis*) and Sparse CCA (*vertical axis*). The dashed line is the 45°-line

Results of the cross-validation scores of Eq. (6) are reported in Fig. 3. For each of the 23 chromosomes, we plot the value of the cross-validation score (0 % trimming) for Robust Sparse CCA (horizontal axis) and Sparse CCA (vertical axis). Results when using 10 % trimming are similar and, therefore, omitted. The cross-validation scores of Robust Sparse CCA are much better than those of Sparse CCA: all points are lying above the 45°-line. For chromosomes 1, 3, 4, and 11, for instance, the cross-validation scores of Robust Sparse CCA are more than 10 times lower than those of Sparse CCA. Since Robust Sparse CCA performs much better, outliers might be present for these chromosomes. Hence, it is safer to use Robust Sparse CCA instead of Sparse CCA.

**Fig. 4:**
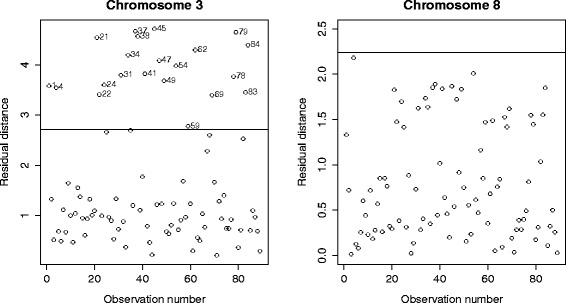
Breast cancer data set: Residual Distance plot for chromosome 3 (*left*) and chromosome 8 (*right*)

The Robust Sparse CCA method yields an interesting way to characterize the outliers. To this end, we create the Residual Distance plot of the residuals $\mathbf {X}{\widehat {\mathbf {A}}} - \mathbf {Y}{\widehat {\mathbf {B}}}$, and this for each of the 23 chromosomes. The Residual Distance plot displays the robust distance of the residuals (vertical axis) versus the observation number (horizontal axis). Points above the horizontal black line are marked as outliers. Results for chromosome 3 and 8 are displayed in Fig. 4, results for the other chromosomes are available upon request. For some chromosomes, like chromosome 3, the difference in cross-validation scores of Robust Sparse CCA and Sparse CCA in Fig. 3 is outspoken, suggesting that outliers might be present. We use the Residual Distance plot (Fig. 4, left panel) to detect which patients are outlying. In the Residual Distance plot of chromosome 3 a lot of patients are marked as outliers. For chromosome 8, on the other hand, the cross-validation scores of Sparse CCA and Robust Sparse CCA are nearly identical, which might suggest that there are no outliers. Looking at the Residual Distance Plot of chromosome 8 (Fig. 4, right panel), no outliers are indeed detected.

## Discussion

Robust Sparse CCA has three important advantages over Robust CCA. (i) Robust Sparse CCA improves model interpretation since only a limited number of variables, those corresponding to the non-zero elements of the canonical vectors, enter the estimated canonical variates (cfr. evaporation application), (ii) if the number of variables approaches the sample size, the estimation precision of Robust CCA suffers, and (iii) if the number of variables exceeds the sample size, Robust CCA can not even be performed. Robust Sparse CCA can still be computed (cfr. nutrimouse and breast cancer application).

The key ingredient of the Robust Sparse CCA algorithm is the sparse LTS proposed by [31]. The choice of the subsample size *h*, see Eq. (2) involves a trade-off between robustness and estimation accuracy. We use *h*=⌊0.75·*n*⌋, as recommended by [31]. This guarantees a sufficiently high estimation accuracy and a good robustness/accuracy trade-off. If the researcher thinks that the proportion of outliers in one of the two data sets is larger than 25 %, one could consider higher values of *h*. Our Robust Sparse CCA algorithm starts by robustly centering each variable using the coordinatewise median. The spatial median (e.g. [37], page 251) could serve as an alternative to the coordinatewise median.

Several questions are left for future research. One could use a joint selection criterion for the number of canonical variate pairs and the sparsity parameter. This would, however, increase computation time substantially. To obtain sparse canonical vectors, we use a Lasso penalty. Other penalty functions such as the Adaptive Lasso [43] could be considered. The Adaptive Lasso is consistent for variable selection, whereas the Lasso is not. Furthermore, we use a regularized version of the LTS estimator. One could also use a regularized version of the S-estimator or the MM-estimator to increase efficiency. Up to our knowledge, however, the sparse LTS is the only robust sparse regression estimator for which efficient code [35] is available.

## Conclusion

Sparse Canonical Correlation Analysis delivers interpretable canonical vectors, with some of its elements estimated as exactly zero. Robust Sparse CCA retains this advantage, while at the same time coping with outlying observations.

Typically, the canonical vectors are based on the sample versions of the covariance matrices. One could think of estimating those covariance matrices with an estimator that is robust and sparse at the same time, and then, to compute the eigenvectors. This approach would result in canonical vectors being robust, however, not sparse. To circumvent this pitfall, we reformulate the CCA problem in a regression framework.

Nowadays, high-dimensional data sets where the researcher suspects contamination to be present are commonplace in genetics. This requires tailored methods such as Robust Sparse CCA to analyze the information they contain.

## Endnotes

^1^ One iteration includes one cycle of estimating ${\mathbf {A}}_{l}^{*}|{\mathbf {B}}_{l}^{*}$ and ${\mathbf {B}}_{l}^{*}|{\mathbf {A}}_{l}^{*}$.

^2^ Less than 5 % of all simulation runs did not reach convergence after 50 iterations. In case of non-convergence, results from the last iteration run are taken.

^3^ We treat the different measurements from the consecutive days as being independent from each other.

## Additional files

Additional file 1 Sparse LTS estimator. (PDF 133 kb)

Additional file 2 Robust Sparse CCA algorithm. (PDF 150 kb)
