# Supplementary material for: Robust sparse canonical correlation analysis
Source: BMC Syst Biol. 2016 Aug 11;10:72. doi: 10.1186/s12918-016-0317-9 (PMC4982144; doi:10.1186/s12918-016-0317-9)
Supplement: Additional file 1 — Sparse LTS estimator. (PDF 133 kb) [file 12918_2016_317_MOESM1_ESM.pdf]

Sparse LTS estimator

Ines Wilms and Christophe Croux

We briefly review the sparse Least Trimmed Squares (LTS) estimator used in the Robust Sparse CCA algorithm to obtain a robust sparse estimate of the canonical vectors. More details can be find in [1] where this estimator was first proposed.

The sparse LTS estimate $\widehat{\boldsymbol{\beta}}$ from a regression of a vector $\mathbf{y}$ on the data matrix $\mathbf{X}$ is defined as

$$\widehat{\boldsymbol{\beta}} = \underset{\boldsymbol{\beta}}{\operatorname{argmin}} \sum_{i=1}^{h} (\mathbf{r}^2(\boldsymbol{\beta}))_{i:n} + h\lambda \sum_{j=1}^{p} |\beta_j|, \tag{1}$$

where the vector of squared residuals is $\mathbf{r}^2(\boldsymbol{\beta}) = (r_1^2, \ldots, r_n^2)^T$ with $r_i^2 = (y_i - \mathbf{x}_i^T \boldsymbol{\beta})^2, i = 1, \ldots, n$. We denote the ordered squared residuals $(\mathbf{r}^2(\boldsymbol{\beta}))_{1:n} \leq \ldots \leq (\mathbf{r}^2(\boldsymbol{\beta})))_{n:n}$. If $\lambda = 0$, the standard Least Trimmed Squares estimator [2] is obtained. The value $h \leq n$ determines the trimming proportion: $(1-h)/n$ percent of the observations are trimmed. The sparse LTS can be interpreted as a trimmed lasso since taking $h = n$ yields the lasso solution [3]. Like the lasso, sparse LTS (i) produces sparse model estimates meaning that some of the components of $\widehat{\boldsymbol{\beta}}$ will be estimated as exactly zero, and (ii) is computable if the number of predictor variables exceeds the subset size h. Unlike the lasso, the sparse LTS estimator is a robust estimator.

The subset size $h = \lfloor (1-\alpha) \cdot n \rfloor$ determines the breakdown point of the sparse LTS which is at least $100 \cdot \alpha\%$. The breakdown point of an estimator is the largest fraction of original observations that can be replaced by arbitrary points without the estimate becoming infinite. We take $\alpha = 0.25$. Then α may be seen as a conservative initial guess of the proportion of outliers in the data. We are rather conservative to ensure that outliers do not impact the results.

To compute the estimator in (1), [1] use an analogue of the iterative FAST-LTS algorithm [4]. This algorithm applies C-steps, starting from different initial values. Assume that an approximated value of (1) is available. Then a C-step consists of computing the lasso estimator from those observations corresponding to the h smallest squared residuals. Each C-step results in a decrease of the sparse LTS objective function. A sequence of C-steps converges to a local minimum in a finite number of steps. This procedure is repeated for a large number of initial values, each of them giving us a local minimum. Among these local minima, the one with the smallest value of the objective function in (1) is selected.

To increase statistical efficiency, a reweighting step is performed that downweights outliers detected by the sparse LTS. An observation is declared to be an outlier if the absolute value of its standardized residual is larger than the 98.75^{th} quantile of the standard normal distribution. The final sparse LTS is the lasso estimator computed from the observations not detected as outliers by the sparse LTS. We use this sparse LTS estimator in the Robust Sparse CCA algorithm from Additional file 2 using the implementation in the `R`-package `robustHD` [5].

References

[1] Alfons, A., Croux, C., Gelper, S.: Sparse least trimmed squares regression for analyzing high-dimensional large data sets. The Annals of Applied Statistics **7(1)**, 226–248 (2013)

[2] Rousseeuw, P.J.: Least median of squares regression. Journal of the American Statistical Association **79**, 871–880 (1984)

[3] Tibshirani, R.: Regression shrinkage and selection via the lasso. Journal of the Royal Statistical Society Series B **58(1)**, 267–288 (1996)

[4] Rousseeuw, P.J., Van Driessen, K.: Computing lts regression for large data sets. Data Mining and Knowledge Discovery **12**, 29–45 (2006)

[5] Alfons, A.: robustHD: Robust Methods for High-dimensional Data. (2014). R package version 0.5.0
