# Supplementary material for: Robust sparse canonical correlation analysis
Source: BMC Syst Biol. 2016 Aug 11;10:72. doi: 10.1186/s12918-016-0317-9 (PMC4982144; doi:10.1186/s12918-016-0317-9)
Supplement: Additional file 2 — Robust Sparse CCA algorithm. (PDF 150 kb) [file 12918_2016_317_MOESM2_ESM.pdf]

Robust Sparse CCA algorithm

Ines Wilms and Christophe Croux

The code to implement the Robust Sparse CCA algorithm is available on http://feb.kuleuven.be/ines.wilms/software. A schematic overview of the algorithm is given below.

Let $\mathbf{X}$ and $\mathbf{Y}$ be two data matrices with the values of the p, respectively q, variables in their columns. Vectors are always column vectors and $\mathbf{1}$ is a vector of ones. $||\cdot||$ stands for the Euclidean norm.

Preliminary steps

(a) $\mathbf{X}_0 := \mathbf{X}_1^* = \mathbf{X} - \mathbf{1}\text{med}(\mathbf{X})^T$, with med($\mathbf{X}$) the coordinatewise median of $\mathbf{X}$

(b) $\mathbf{Y}_0 := \mathbf{Y}_1^* = \mathbf{Y} - \mathbf{1}\text{med}(\mathbf{Y})^T$, with med($\mathbf{Y}$) the coordinatewise median of $\mathbf{Y}$

Alternating regressions: For $l = 1, \ldots, r$

(a) If $l > 1$: Obtain deflated matrices

- For $j = 1, \ldots, p$: obtain residuals $r_{\mathbf{x}_j}$ from LTS regression of jth column of $\mathbf{X}_l^*$ on $\widehat{\mathbf{U}}_l = [\widehat{\mathbf{u}}_1, \ldots, \widehat{\mathbf{u}}_{l-1}]$
- $\mathbf{X}_l^* = [r_{\mathbf{x}_1}, \ldots, r_{\mathbf{x}_p}]$
- For $j = 1, \ldots, q$: obtain residuals $r_{\mathbf{y}_j}$ from LTS regression of jth column of $\mathbf{Y}_l^*$ on $\widehat{\mathbf{V}}_l = [\widehat{\mathbf{v}}_1, \ldots, \widehat{\mathbf{v}}_{l-1}]$
- $\mathbf{Y}_l^* = [r_{\mathbf{y}_1}, \ldots, r_{\mathbf{y}_q}]$

(b) Starting value

- Obtain first robust principal component $\mathbf{z}_{1,l}$ from $\mathbf{Y}_l^*$. This is the first eigenvector of the matrix $\widehat{\mathbf{S}}_{\mathbf{Y}_l^*}$, the estimated spatial sign covariance matrix of $\mathbf{Y}_l^*$.
- Obtain the sparse LTS estimate $\widehat{\mathbf{B}}_l^{\text{init}}$ from regression of $\mathbf{z}_{1,l}$ on $\mathbf{X}_l^*$
- $\widehat{\mathbf{B}}_l^{(0)} = \dfrac{\widehat{\mathbf{B}}_l^{\text{init}}}{||\widehat{\mathbf{B}}_l^{\text{init}}||}$
- $\widehat{\mathbf{v}}_l^{*(0)} = \mathbf{Y}_l^* \widehat{\mathbf{B}}_l^{(0)}$

(c) Iterate until convergence

- Obtain the sparse LTS estimate $\widehat{\mathbf{A}}_l^{*(s)}$ from regression of $\widehat{\mathbf{v}}_l^{*(s-1)}$ on $\mathbf{X}_l^*$
- $\widehat{\mathbf{A}}_l^{*(s)} = \dfrac{\widehat{\mathbf{A}}_l^{*(s)}}{||\widehat{\mathbf{A}}_l^{*(s)}||}$
- $\widehat{\mathbf{u}}_l^{*(s)} = \mathbf{X}_l^* \widehat{\mathbf{A}}_l^{*(s)}$
- Obtain the sparse LTS estimate $\widehat{\mathbf{B}}_l^{*(s)}$ from regression of $\widehat{\mathbf{u}}_l^{*(s)}$ on $\mathbf{Y}_l^*$
- $\widehat{\mathbf{B}}_l^{*(s)} = \dfrac{\widehat{\mathbf{B}}_l^{*(s)}}{||\widehat{\mathbf{B}}_l^{*(s)}||}$
- $\widehat{\mathbf{v}}_l^{*(s)} = \mathbf{Y}_l^* \widehat{\mathbf{B}}_l^{*(s)}$

(d) After convergence, resulting in $\widehat{\mathbf{A}}_l^*, \widehat{\mathbf{B}}_l^*, \widehat{\mathbf{u}}_l^*$ and $\widehat{\mathbf{v}}_l^*$

- $\widehat{\mathbf{A}}_l = \begin{cases} \widehat{\mathbf{A}}_l^* & \textbf{if } l = 1 \\ \text{sparse LTS estimate from regression of } \widehat{\mathbf{u}}_l^* \text{ on } \mathbf{X}_0 & \textbf{if } l > 1 \end{cases}$
- $\widehat{\mathbf{u}}_l = \mathbf{X}_0 \widehat{\mathbf{A}}_l$
- $\widehat{\mathbf{B}}_l = \begin{cases} \widehat{\mathbf{B}}_l^* & \textbf{if } l = 1 \\ \text{sparse LTS estimate from regression of } \widehat{\mathbf{v}}_l^* \text{ on } \mathbf{Y}_0 & \textbf{if } l > 1 \end{cases}$
- $\widehat{\mathbf{v}}_l = \mathbf{Y}_0 \widehat{\mathbf{B}}_l$

Final solution

(a) $\widehat{\mathbf{A}} = [\widehat{\mathbf{A}}_1, \ldots, \widehat{\mathbf{A}}_r]$

(b) $\widehat{\mathbf{B}} = [\widehat{\mathbf{B}}_1, \ldots, \widehat{\mathbf{B}}_r]$

(c) $\widehat{\mathbf{U}} = \mathbf{X}\widehat{\mathbf{A}}$

(d) $\widehat{\mathbf{V}} = \mathbf{Y}\widehat{\mathbf{B}}$
